# Small RNA and Freeze Survival: The Cryoprotective Functions of MicroRNA in the Frozen Muscle Tissue of the Grey Tree Frog

**DOI:** 10.3390/metabo14070387

**Published:** 2024-07-17

**Authors:** Saif Rehman, Kenneth B. Storey

**Affiliations:** Department of Biology, Carleton Univesrity, Ottawa, ON K1S 5B6, Canada; saifrehman@cmail.carleton.ca

**Keywords:** miRNA, *Dryophytes versicolor*, metabolic rate depression, epigenetics, cell signalling, gene ontology

## Abstract

The grey tree frog, *Dryophytes versicolor*, survives whole-body freezing for weeks during cold winter months. Survival in a state devoid of available food, water, or oxygen forces a reliance on metabolic rate depression (MRD) and the reprioritization of bodily functions. This study utilizes next-generation sequencing (NGS) and bioinformatic analyses to characterize changes in the microRNAome of *D. versicolor*. When comparing control to frozen groups, five microRNAs (miRNA) were found to be differentially regulated (miR-143-3p, miR-30e-3p, miR-10a-5p, miR-140-3p, and miR-148a-3p), suggesting that they play key roles in freeze survival. The KEGG and GO analyses of these changes predicted a significant negative enrichment of terms associated with cell proliferation and active metabolism while simultaneously predicting the upregulation of cell signalling terms. These results suggest a fast-acting regulatory role for miRNA in contributing to the reorganization of gene expression and the limitation of energy-expensive processes during MRD in the hind leg skeletal muscle of the frog.

## 1. Introduction

Under extreme cold temperatures, poikilothermic animals are often required to employ one of two strategies to endure the subzero temperatures of winter. Some species use freeze avoidance, employing antifreeze proteins and cryoprotectants to enter a supercooled state [1]. Others employ freeze tolerance that allows ice formation in extracellular or extra-organ fluid spaces while protecting the internal organs from freezing. The grey tree frog, *Dryophytes versicolor* (formerly *Hyla versicolor*), is one of the few vertebrate species that have evolved freeze tolerance, surviving the conversion of up to about 60% of their total body water into extracellular ice. Notably, these frogs produce glycerol as a cryoprotectant that protects their intracellular environment from freezing while overwintering [2,3]. Although dissimilar to other well-studied frog species such as the wood frog, *Rana sylvatica*, that uses glucose as a cryoprotectant, glycerol is the cryoprotectant of choice for most invertebrates, some species of fish, and *D. versicolor*. Ice accumulating in extracellular spaces leads to ischemia and cessation of the heartbeat. In the frozen state, gas exchange and waste export from vital organs (lungs, kidneys, etc.) is also curtailed, leading to a reliance on intracellular fuel reserves and anaerobic metabolism [4,5]. The endogenous fuel reserves in the cells are often too little to sustain energy demand and, hence, extended periods of metabolic rate depression (MRD) are required for survival. Complex regulatory mechanisms elicit the MRD response, including miRNA-mediated gene suppression [6].

Studies in recent years have shown the importance of microRNA (miRNA) in the development, function, and regulation of cell functions and that these small molecules are involved in the regulation of numerous processes, including cell proliferation, differentiation, apoptosis, and angiogenesis. MicroRNAs (miRNAs) are small non-coding RNA molecules, typically 20–25 nucleotides in length, that play crucial roles in regulating gene expression in eukaryotic cells [7]. These small molecules are highly conserved throughout evolutionary history, and many miRNA families have been identified that are shared between distantly related species, indicating their important role in biological processes. The primary function of miRNAs is to regulate the expression of protein-coding gene transcripts by binding to target mRNA molecules, leading to their degradation or translational repression. This process is achieved through complementary base pairing between miRNA and mRNA targets, which results in the suppression of gene expression. Each miRNA type can regulate multiple target mRNAs, and a single mRNA type can also be targeted by multiple different miRNAs, allowing for the complex regulation of gene expression. Because miRNAs play a crucial role in the regulation of gene expression, they have emerged as important tools for understanding biological pathways and networks. By analyzing the miRNA expression profile of cells or tissues, it is possible to infer controls over metabolic pathways and identify potential biomarkers for various diseases [8,9,10]. Additionally, the analysis of miRNA–target interactions can reveal important information about the regulation of specific genes and the signalling pathways that they are involved in.

Since miRNAs have such widespread use in the regulation of pathways under normal conditions, there is a strong implication that they would also have key roles in navigating extreme stress situations. The grey tree frog (*Dryophytes versicolor*) can survive extended periods of time without food or normal levels of oxygen, and enduring subzero temperatures in a frozen state. Hence, it is an ideal organism to highlight the changes required to survive such ordeals. Previous studies of miRNA from *D. versicolor* highlighted changes in miRNA expression in the liver and miRNA action in downregulating energy-expensive pathways [11]. Since the liver is an internal organ with vital functions that support survival, it is likely that the liver also utilizes different survival mechanisms and pathways than does muscle tissue. As muscle is a superficial tissue that experiences no notable function when frozen for extended periods of time, its complete return to normal function upon thawing is even more profound. To build a better picture of how these frogs can survive for extended periods of time while frozen, and to understand the implications this could have on current biotechnology, an analysis of differentially expressed miRNAs is an important research target. Utilizing small RNA-seq and a comprehensive bioinformatics strategy, with elements of systems biology, the present study aimed to characterize the metabolic significance of differential miRNA expression in the muscle of frozen grey tree frogs in comparison to active controls. By integrating data gleaned through in silico analysis, this approach enables a discussion of a wide range of pathways and gauging overall trends. Identifying the regulatory changes within muscle required to transition to a hypometabolic, pro-survival frozen state could provide insight into the most relevant adaptations to extreme environmental stresses. This broad analysis of differential miRNA is designed to help future studies better target the downstream processes of importance to stress survival.

## 2. Materials and Methods

### 2.1. Animal Treatments and Tissues

Adult male grey tree frogs, *D. versicolor*, weighing 6–11 g (mean = 7.883, sd = 1.197) were collected from breeding ponds in the Ottawa area (Ottawa, ON, Canada) as previously described [12,13]. The frogs were acclimated to temperatures of 5 °C for 2 weeks in plastic containers lined with damp sphagnum moss and without feeding. Control frogs were randomly selected from this group. Other groups of 4–5 frogs were placed in trays lined with damp paper towels and placed in an adjustable temperature incubator, first at −4 °C for 45 min to trigger ice nucleation when the frogs cooled below 0 °C. Subsequently, the temperature was raised to −2.5 °C and a 24 h freeze exposure was initiated. The euthanasia of both control and frozen frogs was performed via double-pithing, followed by the prompt dissection of selected tissues, including hind leg skeletal muscle. Tissues were flash-frozen in liquid nitrogen and stored at −80 °C until use. Animal care, experimentation, and euthanasia procedures adhered to the guidelines of the Canadian Council on Animal Care and had the prior approval of the Carleton University Animal Care Committee (protocol no. 13683).

### 2.2. Total RNA Extraction

Total RNA was isolated from the leg skeletal muscle of control (n = 4) and frozen (n = 3) frogs as previously described [14,15]. Approximately 50 mg of frozen muscle was crushed by a mortar and pestle under liquid nitrogen and then homogenized in 1 mL of TRIZOL reagent (Invitrogen; Cat. # 15596-018) using a Polytron PT1200 homogenizer (Kinematica, Werkstrasse, Switzerland). A 200 μL aliquot of chloroform was then added to each sample followed by centrifugation at 10,000× *g* at 4 °C for 15 min. Total RNA settled in the upper aqueous phase and was transferred to a new tube, and then the RNA was precipitated by adding isopropyl alcohol (500 μL) and incubating at room temperature for 10 min. Samples were then centrifuged at 10,000× *g* for 15 min at 4 °C followed by removal of the supernatant. RNA pellets were then washed twice with 70% ethanol and air-dried for 10 min. Next, RNA pellets were resuspended in 50 μL of RNAse-free water and then assessed for RNA concentration and purity using a Take3 micro-volume quantification plate and a PowerWave HT spectrophotometer. Following determination that the 260/280 nm ratio indicated RNA > 1.8, an aliquot of RNA sample was confirmed for integrity on a 1% agarose gel and stained with SYBR Green. The final RNA samples were then isolated and standardized to a concentration of 1 µg/µL followed by freezing and storage at −80 °C until use.

### 2.3. Small RNA Sequencing

RNA samples from the muscle of both control and 24 h frozen *D. versicolor* were sequenced by the Quebec Centre de Recherche (Laval, QC, Canada). Prior to microRNA library construction, RNA quality was assessed using a Qubit Fluorometer (Invitrogen, Carlsbad, CA, USA) and an Agilent Bioanalyzer 2100 system (Agilent Technologies, Santa Clara, CA, USA). Subsequently, small RNA cDNA libraries were constructed, validated with the Bioanalyzer, and sequenced using an Illumina NovaSeq 6000 platform to produce single-end 100 base reads. All sequencing data are available on the Sequence Read Archive (SRA; BioProject ID: PRJNA1078871).

### 2.4. Data Processing

Raw read data were processed as outlined in previous papers using the RBioMIR pipeline [16]. CutAdapt was used to trim the adapters from all reads followed by the Phred quality scores being assessed by FastQC, which was used to validate adapter removal and remove reads with scores below 30 using fastq-mcf. A negative reference file compiled from the Rfam and piRNABank databases was used to filter out non-miRNA small RNA species (e.g., rRNA, tRNA, snRNA, snoRNA, and piRNA) and Bowtie was used to remove reads aligned to the negative reference file. The resulting miRNA reads were aligned to a positive reference file of mature miRNA sequences from miRbase using Bowtie. A seed sequence length parameter of 20 nucleotides was set with only perfect sequence matches being reported. Mature miRNA read counts with more than four reads were sorted and determined using SAMtools software and Unix command line tools for filtered reads. Finally, all filtered read counts were standardized using the Voom method as previously described [17,18].

### 2.5. Differential Expression Analysis

The differential expression of miRNA data from the control and frozen samples was analyzed using the linear models for microarray data (limma) R package with linear-model-fitting empirical Bayesian testing [19]. Differentially expressed miRNAs were considered significant if the false discovery rate (FDR)-adjusted *p* < 0.05 and a fold change was as follows:

|FC| ≥ 1.5(1)

### 2.6. Gene Set Analysis

Gene set analysis was carried out using the RBiomirGS R package [16], implementing logistic regression-based analysis for significantly enriched gene ontology (GO) terms and Kyoto Encyclopedia of Genes and Genomes (KEGG) pathways [20]. Fully conserved human miRNA orthologs were used as substitutes for *D. versicolor* due to the lack of a sequenced genome for this species. RBiomirGS was used to calculate a miRNA score (S_miRNA_) for each miRNA using the formula:

SmiRNA = −log_10_ *p* × *signum*(log_2_ *FC*) (2)

where *p* is the FDR-adjusted *p*-value and *FC* is the fold-change of the miRNA between the control and frozen frogs from the differential expression analysis. Additionally, a mRNA score (*S_mRNA_*) for each mRNA target was computed as:

(3) $$S_{\mathrm{mRNA}}=-\sum_{i=1}^{n}S_{\mathrm{miRNA}}^{i}$$

where *n* is the number of miRNAs targeting a given mRNA, and $S_{\mathrm{miRNA}}^{i}$ is the *S_miRNA_* of the *i*th targeting miRNA. Enriched gene sets with FDR-adjusted *p*-values ≥ 0.05 and corresponding model coefficients for each GO term and KEGG pathway were determined by RBiomirGS using the calculated *S_mRNA_* of mRNAs. Positive model coefficients indicated decreased negative regulation by microRNA, whereas negative coefficients indicated increased negative regulation by microRNA in the frozen group compared to the control group. The estimated model coefficients and standard error were calculated using logistic regression to determine statistical significance [16].

## 3. Results

### 3.1. Analysis of Differentially Expressed miRNAs

**Figure 1:**
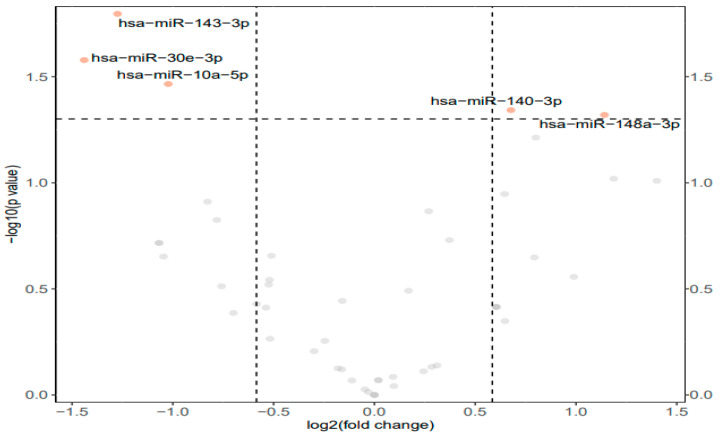
Differential expression analysis of conserved miRNAs in the muscle tissue of *D. versicolor*. Volcano plot of the differential expression of conserved miRNAs showing −log_10_(*p* value) versus the log_2_(fold change) for individual miRNAs in muscle from frozen frogs versus the control group. Points in orange represent miRNAs that are significantly different from the control (>1.5-fold change and *p* < 0.01), grey points represent non-significant miRNAs.

From the total group of forty-nine mature miRNAs evaluated, five miRNAs were identified as differentially expressed (*p* < 0.05 and fold change >1.5) in the hind leg skeletal muscle of frozen *D. versicolor* as compared with control frogs. Of these, three miRNAs were downregulated whereas a significant upregulation of two miRNAs was observed in samples from frozen frogs. A volcano plot was used to visualize the differential expression of miRNAs from the muscle tissue of control and frozen frogs (Figure 1). The downregulated miRNAs were miR-143-3p, miR-30e-3p, and miR-10a-5p. The upregulated miRNAs were miR-140-3p and miR-148a-3p.

### 3.2. Gene Set Analysis

**Figure 2:**
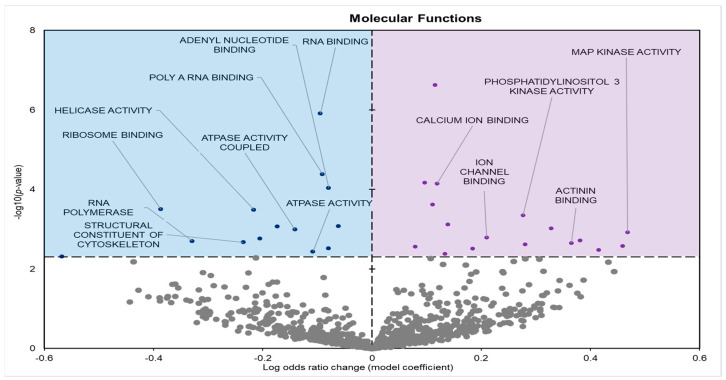
Volcano plot demonstrating the enrichment of GO molecular function (GO MF) through a logistic regression-based gene set analysis of the mRNAs predicted to interact with differentially expressed (DE) miRNAs in frog muscle during freezing. Enriched terms are considered significantly altered if the FDR adjusted *p*-value is <0.05 and fold change >1.5. Negative model coefficients depicting decreased expression are shown as blue circles in the blue-shaded box (left), whereas coefficients depicting increased expression are shown by purple dots in the purple-shaded box (right).

Gene enrichment analyses of GO terms for molecular functions (GO MF) identified 33 significantly affected terms in the leg skeletal muscle of frozen frogs: 16 negative model and 17 positive model coefficients (Figure 2).

**Figure 3:**
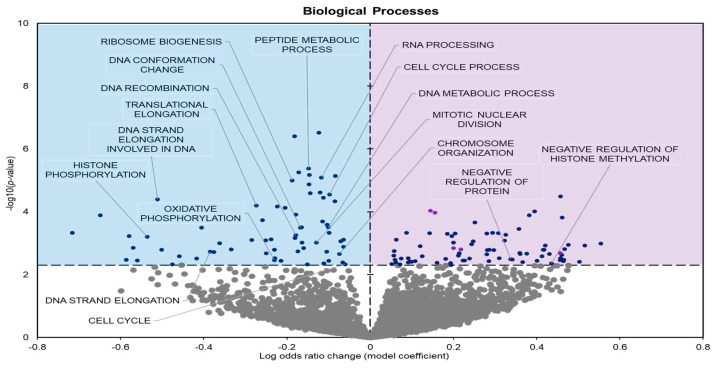
Volcano plot demonstrating the enrichment of GO biological processes (GO BP) through a logistic regression-based gene set analysis of the mRNAs predicted to interact with DE miRNAs in frog muscle during freezing. All other information as in Figure 2.

Biological processes (GO BP) showed 141 significantly enriched terms when comparing controls to frozen groups; 69 had negative model coefficients whereas 72 had positive coefficients (Figure 3).

**Figure 4:**
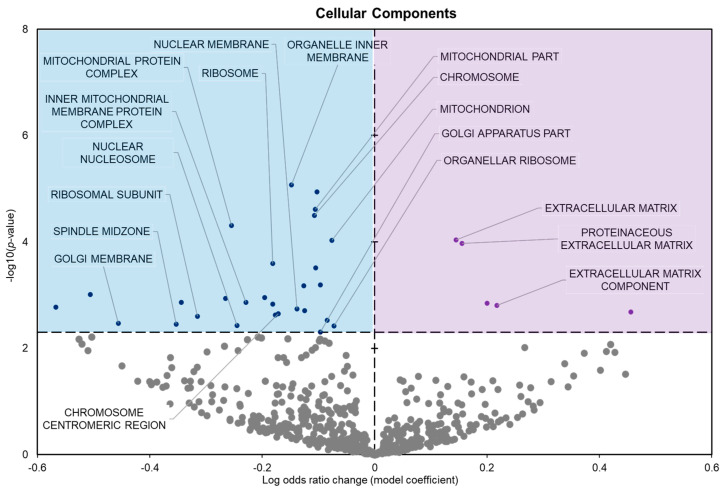
Volcano plot demonstrating the enrichment of GO cellular components (GO CC) through a logistic regression-based gene set analysis of the mRNAs predicted to interact with DE miRNAs in frog muscle during freezing. All other information as in Figure 2.

The cellular components showed 34 significantly enriched terms with 29 negative model coefficients and 5 positive model coefficients (Figure 4). 

**Figure 5:**
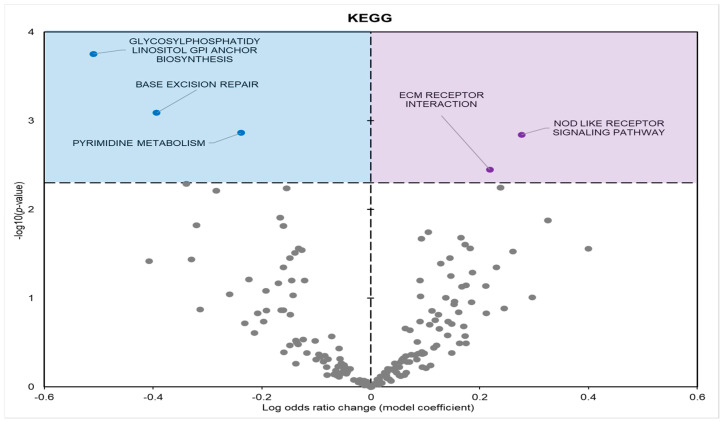
Volcano plot demonstrating the enrichment of KEGG pathways through a logistic regression-based gene set analysis of the mRNAs predicted to interact with DE miRNAs in frog muscle during freezing. The KEGG pathway is considered significantly altered if the FDR adjusted *p*-value < 0.05. All other information as in Figure 2.

KEGG enrichment analysis found five statistically enriched pathways in samples from frozen frogs; three with positive model coefficients and two with negative model coefficients (Figure 5). GO and KEGG term enrichment was predominantly focused on processes related to stimulus detection, metabolism, cell cycle, and ribosomal regulation.

## 4. Discussion

An examination of the response to freezing in *D. versicolor* muscle tissue identified 49 miRNAs, with 5 of these displaying unique expression patterns under freezing stress (Figure 1). An increase in miRNA expression highlights the negative regulation of targeted pathways whereas a decrease in miRNA expression indicates a reduction/lack of inhibition on targets. It is important to note that this does not directly imply an increase in gene/protein expression, but just that a given pathway is not increasingly inhibited by miRNA expression. In this study, the upregulated miRNAs included miR-140-3p and miR148a-3p whereas the downregulated miRNAs included miR-10a-5p, miR-30e-3p, and miR-143-3p. As the downstream effects of miRNAs are the most important, the potential effects of these miRNAs were assessed via gene set analyses which covered GO terms and KEGG pathways. It was found that the miRNAs with significantly altered expression were largely linked to cell cycle regulation, gene expression, ribosomal regulation, histone modifications, and metabolism (Table A1). Since the miRNAs found to be over-expressed during freezing do not exclusively depict a strong inhibitory effect on these pathways, the elevated miRNA levels likely contribute to an overall regulation of pathways rather than just a single inhibitory one. As this is a novel study on miRNA expression in *D. versicolor* leg muscle, there are no comparisons that can be made to previous findings.

During freezing, terms heavily associated with the cell cycle and active cell division were significantly downregulated in *D. versicolor* muscle. The GO MF terms facing downregulation included helicase activity and cytoskeleton constituents, vital functions needed for DNA replication and cell proliferation (Figure 2) [21]. GO BP predicted significant reductions in processes including the cell cycle, DNA strand elongation, DNA recombination, and mitotic nuclear division among other related terms (Figure 3). GO CC also predicted significant reductions in terms related to the cell cycle with negative coefficients on cellular components such as the spindle midzone, chromosome centromeric region, and chromosome (Figure 4). GPI–anchor biosynthesis was one of the few KEGG terms that were significantly negatively enriched. However, its relevance to dynamic changes in cell proliferation through signal transduction is not dissimilar from the effects of other negatively enriched GO terms (Figure 5) [22]. These pathways and processes were predicted to be strongly suppressed due to miRNA activity and suggest a decrease in cell division and cell proliferation in the frozen state.

Two main reasons for processes related the cell cycle being downregulated in the frozen state can be postulated: (a) preventing disease during prolonged winter freezing, and (b) conserving energy. It is well documented that under extreme stress conditions, organisms conserve as much energy as possible via metabolic rate depression (MRD) [23,24]. In *D. versicolor*, and animals that undergo comparable stresses like *Rana sylvatica*, a steady decrease in ATP production often warrants an equally significant suppression of ATP-expensive processes. This prioritizes the use of low energy stores in crucial pro-survival pathways. As cell division has little to contribute to freezing survival and can also strip vital resources from cells already reliant on low fuel/energy stores, the process of downregulation is an effective means of maintaining MRD [25]. Previous studies corroborate evidence of cell cycle arrest/suppression under stress conditions that similarly expose cells to nutrient, oxygen, and/or hydration limitations [26,27,28]. By limiting cell division via reductions in cell cycle-promoting pathways and processes, a two-pronged result of saving energy and avoiding a diseased state while frozen can more easily be achieved.

The expression of miRNAs in *D. versicolor* muscle during freezing was largely associated with gene transcription and translation terms. GO analyses consistently revealed the downregulation of terms associated with active transcription and translation. GO MF analyses predicted the downregulation of RNA polymerase, poly-A RNA binding, RNA-binding functions, and ribosome binding, terms strongly associated with gene expression (Figure 2) [29,30,31]. GO BP terms relevant to gene expression were also strongly negatively affected, including ribosome biogenesis and protein maturation (Figure 3). Related GO cell cycle terms that were downregulated included components of the ribosome and ribosome subunit (Figure 4).

Many terms were found to be associated with the downregulation of gene transcription and protein translation. RNA polymerases are enzymes required for gene transcription and a decrease in their function would suggest a limitation on actively transcribed genes. Similarly, RNA binding plays major roles in transcription factor (TF) function, with its downregulation implying an added regulatory form of negative control on active transcription by miRNAs [30]. Viewing gene expression from the scope of translation shows a similar scenario. GO terms related to ribosomes, which are largely responsible for protein synthesis, were negatively enriched across biological processes and cellular components. Given the energetic cost of both ribosome biosynthesis and protein synthesis, the negative regulation of these processes is an effective means of conserving energy while in a state of hypometabolism in response to stress [5,24,32,33]. The predicted downregulation of terms associated with active gene expression support findings that miRNA may play important roles in regulating the MRD response in freezing.

Interestingly, studies of diverse tissues in similar organisms found dissimilar results. Studies of miRNA expression in *Scaphiopus couchii* and *Xenopus laevis* heart in response to a significant loss of body water under dry conditions (these species undergo summer estivation) revealed the upregulation of both protein translation and transcription terms [14,15]. Since the heart is a core organ that is still active and vital to survival across a variety of stresses, a more superficial tissue like muscle understandably has a more complete downregulation of energy-expensive processes. These notable differences in expression profile increase evidence of the tissue-specific regulation of gene expression during MRD, in this case caused by miRNA action. 

GO BP predicted terms associated with histones, namely histone methylation and phosphorylation, to be negatively enriched during freezing (Figure 3). Post-translational modifications (PTMs) of histones are regulatory mechanisms that can control gene expression. At a transcriptional level, the modification of specific residues influences chromatin structure. This can play an important role in metabolic control and cell survival when facing environmental stresses.

Histone phosphorylation is often associated with the relaxation of chromatin via various means. The phosphorylation of H3S10ph influences H3 acetylation, a transcription activator. H3S28ph in combination with H3K27ac plays roles in gene activation [34]. The phosphorylation of T41 on H3 has also been stated to lead to transcriptional activation via inhibiting chromatin binding to heterochromatin protein 1 α (HP1α). Adenosine monophosphate-activated protein kinase (AMPK) also activates transcription through association with H2Bph at S36 [35,36]. Although histone phosphorylation on four residues of H3 (T3, S10, T11, S28) is associated with chromatin compaction, its effects are largely involved in chromatin relaxation alongside its regulation of overall gene expression.

The functional outcome of histone modifications often depends on the residue being modified. In the case of methylation, the number of methyl groups attached (mono-, di-, tri-methylated) is also key to the modification on gene expression. H3K4me3 is associated with active transcription and H3K4me1 is associated with an enhancer function, whereas H3K27me3 is linked to a repressed chromatin state [37]. The predicted negative regulation imposed by miRNA on histone modifications is complex, as histones play a large role in gene regulation as well. Although an overarching conclusion of decreased gene expression can be drawn from the present predictive results, possibilities such as the negative regulation of modifications that downregulate gene expression require further in-depth study.

A crucial target of miRNA regulation is cell signalling pathways. Predicted upregulated GO MF terms included phosphatidylinositol kinase (PI3K) and mitogen-activated protein kinase (MAPK) activities (Figure 2). Positive enrichment of the KEGG term “Nod-like receptor (NLR) signalling pathway” is also of importance, since the stimulation of the NLR signalling pathway has been found to activate MAPKs and other signal-transducing enzymes [38,39].

PI3K and MAPK coordinate extracellular cues to cells, leading to a large variety of downstream effects, and play an important role in cell fate [40,41,42]. As crucial elements in signal transduction pathways, these two kinases play major roles in controlling cell survival, division, and metabolism [43]. These signalling pathways can also have varying effects depending on the stimulus. For example, depending on the stimulus and the cell type under stress, MAPK pathways can be either pro-survival or pro-apoptotic [44].

Past studies have made it clear that there are countless stress factors associated with freezing survival that can induce apoptosis in cryopreserved cells, including vascular damage by ice formation, energy limitations, and osmotic imbalance. The involvement of apoptosis in cryopreservation failure has also been widely reported [45]. It is plausible that a lack of miRNA inhibition on MAPK and PI3K function during freezing is an important means by which to promote cell survival. One study found that the activation of a MAPK subfamily, p38MAPK, was protective against oxidative stress-induced apoptosis, which is experienced in a cryoprotective state [46]. JNK (a member of the MAPK subfamily) can also exhibit anti-apoptotic functions via the protein phosphorylation of Bcl-2-associated death promoter (BAD) and preventing BAD-induced apoptosis [44]. PI3K activation is linked to the phosphorylation of AKT and is highly conserved [47]. The effect of AKT is based largely on its ability to phosphorylate transcription factors that promote cell survival genes, such as nuclear factor κB (NFκB), and its negative regulation of transcription factors that promote cell death genes (e.g., forkhead box proteins) [47]. Like the JNK/MAPK subfamily, AKT mediated phosphorylation of BAD also inhibits its apoptotic effects [48]. NLR signalling pathways, aside from the activation of MAPK, similarly activate downstream molecules that could be involved in promoting cell survival, such as NFκB [49].

Despite the overarching downregulation of processes in *D. versicolor* muscle, the lack of inhibition on cell signalling pathways implies active cell regulation in frozen muscle tissue cells. The suppression of apoptosis via cell signalling pathways is an effective means of aiding freezing survival and maintaining cell viability over long periods of time. This could be especially important in hind leg muscle tissue as it is largely inactive throughout freezing episodes and susceptible to dysfunction when thawed. This study thereby highlights the genes in regulatory pathways prominently affected by miRNA for future targeted downstream analyses.

## 5. Conclusions

The results herein demonstrate a subset of five miRNA molecules (miR-140-3p, miR148a-3p, miR-10a-5p, miR-30e-3p, and miR-143-3p) that are predicted to be the most important to the freeze response in skeletal muscle. Bioinformatic analysis predicted a significant downregulation of pathways associated with the cell cycle, active protein translation, and targets early in gene expression pathways. The recurring theme of miRNA suppression of energy-expensive processes is likely an effective means of conserving vital fuel/energy stores until a return to normal muscle function is permitted by environmental conditions. Pathways associated with cell signalling and stimulus detection were predicted to be upregulated even during a frozen state in muscle tissue. The lack of miRNA inhibition of the MAPK and PI3K cell signalling processes highlights the role of cell fate determination in freezing, with a focus on anti-apoptosis to prolong cell survival. An overall downregulation of histone modifications by miRNA interference warrants further study given the complexity of the current findings. This study was able to identify key miRNAs and their targets that are involved in muscle tissue responses to aid freeze survival. These findings highlight new avenues for the freeze tolerance studies of tissues from varying stress-tolerant organisms and indicate the importance of miRNA on global MRD during the freezing of freeze-tolerant species.

## Data Availability

All sequencing data are available on Sequence Read Archive (SRA; BioProject ID: PRJNA1078871). The data that support the findings of this study are available from the corresponding author upon reasonable request.

## Appendix A

**Table A1:** Significantly enriched GO term statistical values.

Category	GO Term	Coefficient	*p*-Value
Molecular Functions	Helicase activity	−0.2165	0.000327
	RNA polymerase	−0.32913	0.00201
	MAPK activity	0.468236	0.00120
Biological Processes	Ribosome biogenesis	−0.18786	0.0000102
	Cell cycle	−0.05995	0.004741
	DNA recombination	−0.17865	0.000559
Cellular Components	Chromosome	−0.10697	0.0000320
	Ribosome	−0.18159	0.000256
	Spindle midzone	−0.4559	0.00355
